# New paths in pediatrics - The implementation of a Unit of Integrative Pediatrics at the Institute of Children and Adolescents, University of Sao Paulo

**DOI:** 10.6061/clinics/2021/e3351

**Published:** 2021-11-17

**Authors:** Paula Lage Pasqualucci, Rita Tiziana Verardo Polastrini, Georg Seifert, Magda Carneiro-Sampaio, Vicente Odone, Angélica Carreira dos Santos

**Affiliations:** I Departamento de Pediatria, Faculdade de Medicina FMUSP, Universidade de Sao Paulo, Sao Paulo, SP, BR.; II Instituto da Crianca e do Adolescente (ICr), Hospital das Clinicas HCFMUSP, Sao Paulo, SP, BR.; III Charité - Universitätsmedizin Berlin, Berlin, Germany.

The prevalence rates of chronic and complex pediatric health conditions have increased dramatically in recent decades, and a change in the healthcare delivery model seems necessary to meet the latest challenges faced by children, families, and healthcare providers. Integrative health is defined as patient-centered care that focuses on the whole person and uses the most appropriate therapies to successfully achieve wellness, including evidence-based complementary practices. Complementary health practices are defined as evidence-based practices developed outside of conventional Western medicine (1).

Integrative pediatrics develops and promotes this approach within the field of pediatrics. It reflects a “redesign” of the pediatric healthcare model to that of a patient-family-centered care model and treats the patient-family unit as a whole—incorporating the mind, body, and spirit—in a frame of wellness promotion rather than in a “disease-centered” approach. Integrative pediatrics aims to offer the best available care for children, considering complementary practices and conventional health in integration and partnership with the multiprofessional teams and all pediatric specialties (2).

According to the American Academy of Pediatrics, results from the 2012 National Health Interview Survey (NHIS) revealed that the prevalence of children using complementary therapies remained approximately 50% among children living with chronic illness. In the same survey, most pediatricians agreed they should provide patients with information about all potential treatment options, but reported they had little or no knowledge of complementary therapies and integrative health (3).

In 2012, 16 accredited medical schools in North America adopted academic integrative pediatric programs. All programs provided clinical services, and 75% provided both inpatient and outpatient services. The most common conditions addressed were cancer, mental health, and chronic pain (4). 

Most pediatric hospitals worldwide have not yet institutionalized integrative pediatric programs. In Germany, for example, a model project has been initialized in three different pediatric centers, and over three years, pediatric complementary therapies have been integrated into routine pediatric conventional care, and a new integrative health department has been established. The modalities applied included Traditional Chinese Medicine (TCM), relaxation, hypnosis, reflexology, aromatherapy, homeopathy, yoga, and herbal medicine. The German experience shows that with motivated staff, structured planning, and adequate funding, implementation of integrative health in a children’s hospital can be successful (5).

As part of a pioneering plan, the Institute of Children and Adolescents of the Hospital das Clinicas of the Faculty of Medicine, University of Sao Paulo, in partnership with the Charité - Universitätsmedizin Berlin, founded a Unit of Integrative Pediatrics (UPI) in 2017 and completely implemented the unit model in 2021, after three years of pilot experience. The current UPI model is philanthropically financed and consists of a team of three coordinators, six healthcare professionals, three researchers, and one database assistant. The unit’s main goal is to develop an updated healthcare model for pediatric patients with chronic and complex conditions, emphasizing health and wellness promotion and the combined use of conventional and complementary therapies. Previous pilot experience emphasized the importance of keeping the integrative team small and well-integrated with the institution’s multidisciplinary team (6).

The unit’s clinical framework is intended to deliver systematic patient-centered care focused on four major clinical conditions: mental health promotion, child-family unit care, medication use reduction, and patient self-care skills promotion. The majority of patients struggle with inconsistent attention from the conventional health system to deal with these conditions, and this gap is a considerable challenge for healthcare professionals.

To meet our clinical framework, the best available therapies for children embracing the mind, body, and spirit were chosen to be incorporated into our institution’s routine care: music therapy and anthroposophic external therapy for inpatient services, and a mindfulness-based program for outpatient services. The emphasis was on choosing non-invasive and non-pharmacological interventions with good practical applicability while balancing safety and efficacy concerns (7).

Research and educational opportunities in pediatric integrative health are abundant. The opportunities must go beyond the efficacy and mechanism of action investigation studies and privilege real-life experiences with pragmatic clinical trials and implementation research designs. Our scientific goals are to create protocols applicable to different hospitals in Brazil that aim to develop a different model of care and contribute scientifically to integrative pediatrics, implementation sciences, and qualitative research.

We are convinced that the adoption of systematic patient-centered care that focuses on wellness and health promotion and treats the child-family unit as a whole is an inevitable shift in the healthcare paradigm and a necessary path for the future of optimal pediatric healthcare, especially for pediatric patients with chronic and complex health conditions.

## AUTHOR CONTRIBUTIONS

Pasqualucci PL and Santos AC contributed to the conception and drafted the manuscript. Polastrini RTV, Seifert G, Carneiro-Sampaio M and Odone Filho V contributed to the conception and critically reviewed the manuscript. All authors read and approved the final version of the manuscript.
